# Calcium-independent disruption of microtubule dynamics by nanosecond pulsed electric fields in U87 human glioblastoma cells

**DOI:** 10.1038/srep41267

**Published:** 2017-01-24

**Authors:** Lynn Carr, Sylvia M. Bardet, Ryan C. Burke, Delia Arnaud-Cormos, Philippe Leveque, Rodney P. O’Connor

**Affiliations:** 1 XLIM Research Institute, UMR CNRS No 7252, University of Limoges, Faculty of Science and Techniques, 123 Avenue Albert Thomas, 87060 Limoges, France

## Abstract

High powered, nanosecond duration, pulsed electric fields (nsPEF) cause cell death by a mechanism that is not fully understood and have been proposed as a targeted cancer therapy. Numerous chemotherapeutics work by disrupting microtubules. As microtubules are affected by electrical fields, this study looks at the possibility of disrupting them electrically with nsPEF. Human glioblastoma cells (U87-MG) treated with 100, 10 ns, 44 kV/cm pulses at a frequency of 10 Hz showed a breakdown of their interphase microtubule network that was accompanied by a reduction in the number of growing microtubules. This effect is temporally linked to loss of mitochondrial membrane potential and independent of cellular swelling and calcium influx, two factors that disrupt microtubule growth dynamics. Super-resolution microscopy revealed microtubule buckling and breaking as a result of nsPEF application, suggesting that nsPEF may act directly on microtubules.

Traditional cytotoxic chemotherapies are associated with severe side-effects and new generation targeted therapies can fail due to drug resistance. High powered, nanosecond duration pulsed electric fields (nsPEF) have been proposed as a minimal side-effect, electrical cancer therapy that is unlikely to result in resistance. Glioblastoma multiforme (GBM) is an incurable brain cancer showing resistance to surgery, radiotherapy and chemotherapy^1^. The need for an effective treatment for GBM, and its previously demonstrated sensitivity to pulsed electric fields^2^, makes it of interest for targeting by nsPEF.

Studies have demonstrated that nsPEF induce cell death by apoptosis and necrosis *in vitro* and reduce the size of tumours both in animal models and in humans^3^,^4^,^5^,^6^,^7^,^8^. The effect of nsPEF on cells is characterized by nanoporation of the plasma membrane^3^,^9^,^10^,^11^,^12^, rapid phosphatidylserine externalisation^6^,^13^,^14^, transient spikes in intracellular calcium concentration that are proportional to pulse intensity^3^,^15^,^16^,^17^, loss of mitochondrial membrane potential (ΔΨ_m_)^18^,^19^ and cellular swelling and blebbing^12^,^20^,^21^. Apoptosis following nsPEF treatment can be either dependent or independent of caspase activation^6^,^7^,^13^. Whilst nsPEF induced apoptotic death has been well studied, the mechanism whereby nsPEF triggers apoptosis remains unclear.

Microtubules are hollow, cylindrical, structures composed of repeating α and β heterodimers of the protein tubulin. Forming part of the cell cytoskeleton, microtubules are highly dynamic structures subject to constant lengthening and shortening. In interphase cells they are nucleated in microtubule-organizing centres and grow out towards the cell periphery. Depolymerisation of the interphase microtubule network is an intrinsic, early event in the execution phase of normally occurring apoptosis, aiding phagocyte attachment^22^ and the release of microtubule sequestering proapoptotic proteins^23^.

Due to the polarity of their protein structure and charge, it has been shown *in vitro* that purified microtubules will align with an electric field^24^,^25^ and that, in cells, electric fields can disrupt their polymerization^26^. Given these properties, we hypothesized that nsPEF might have a direct effect on microtubules.

In this study we have used live-cell imaging of U87 glioblastoma cells to visualise both microtubules, in cells expressing tubulin-RFP, and their growth dynamics, in cells expressing the microtubule plus end tracking protein EB3-GFP. EB3-GFP binds only to the tips of growing microtubules and produces a characteristic comet-like fluorescence and therefore gives an indication of the number of polymerising microtubules and their growth trajectories. We demonstrate that 100, 10 ns pulses delivered at a frequency of 10 Hz cause a rapid disruption of microtubule growth and we show that this effect is independent of increases in intracellular calcium levels and cellular swelling. Super-resolution microscopy revealed microtubule buckling and breaking as a result of nsPEF application, suggesting a possible mechanism. We confirm also that a loss of ΔΨ_m_ closely follows the disruption of microtubule growth suggesting a link between the two events.

## Results

### Application of nsPEFs to glioblastoma cells cause a dose dependent uptake of YO-PRO-1

To determine the effect of different dosing strategies on U87 cells we subjected them to increasing numbers of pulses and increasing pulse repetition rates. YO-PRO-1 is a dye that is excluded from cells with intact plasma membranes and by measuring its uptake into these cells we were able to determine the extent of membrane poration.

**Figure 1:**
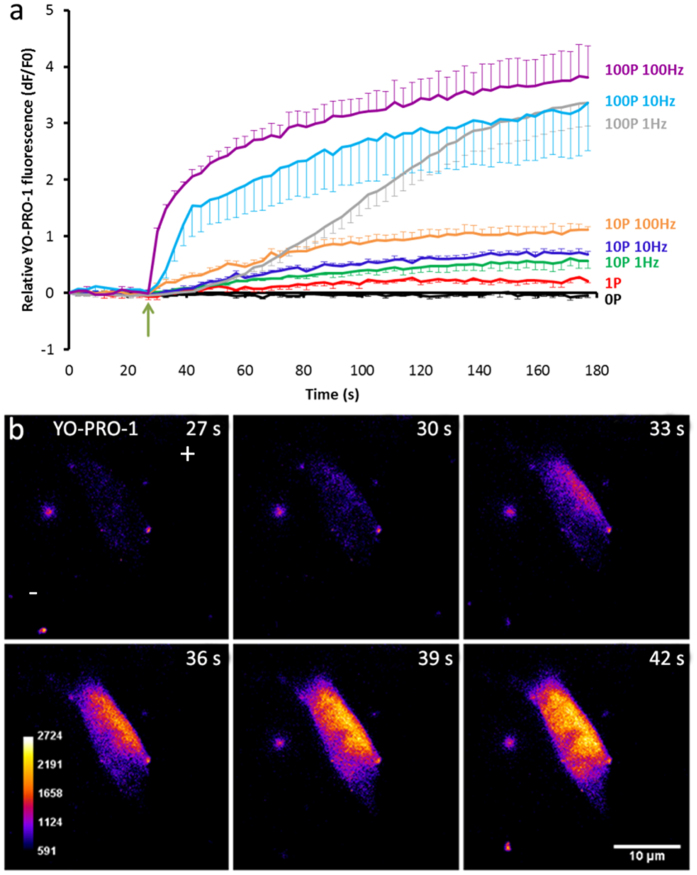
Temporal and spatial uptake of YO-PRO-1 as a function of pulse number and frequency. (**a**) Change in fluorescence over time from live cell imaging experiments on YO-PRO-1 uptake into U87 cells following application of 10 ns pulses at varying pulse numbers and repetition rate frequencies. The start of pulse application is represented by a green arrow. Error bars show S.E. 0P n = 5, 1P n = 4, 10P 1 Hz n = 4, 10P 10 Hz n = 7, 10P 100 Hz n = 4, 100P 1 Hz n = 7, 100P 10 Hz n = 4, 100P 100 Hz n = 4. (**b**) Representative live cell images of YO-PRO-1 cellular uptake following 100, 10 ns pulses at 10 Hz. The pulse train was applied just after the image capture at 27 seconds. The images are pseudocoloured for contrast and the colour calibration bar represents arbitrary units of fluorescence. The electrode orientation is marked by the + and − in the first images of the series.

We observed that higher frequencies of pulse application caused a more rapid uptake of dye, likely due to a shorter amount of time being needed to apply the same amount of pulses, and that higher pulse numbers resulted in more overall dye uptake. In all cases the uptake of dye plateaued before the end of the imaging period suggesting the resealing of membrane pores (Fig. 1a). For our subsequent experiments we chose to apply 100 pulses at 10 Hz as it was the minimal dosing strategy required to cause substantial, rapid membrane poration. We considered membrane poration as important since the previously observed depolymerisation of microtubules following nsPEF treatment^27^ has been attributed to an influx of extracellular calcium, a microtubule destabiliser. YO-PRO-1 entry into the cell was not uniform and instead entered as a polarized wave before diffusing across the cell (Fig. 1b).

### nsPEFs disrupt the microtubule network and microtubule growth dynamics

**Figure 2:**
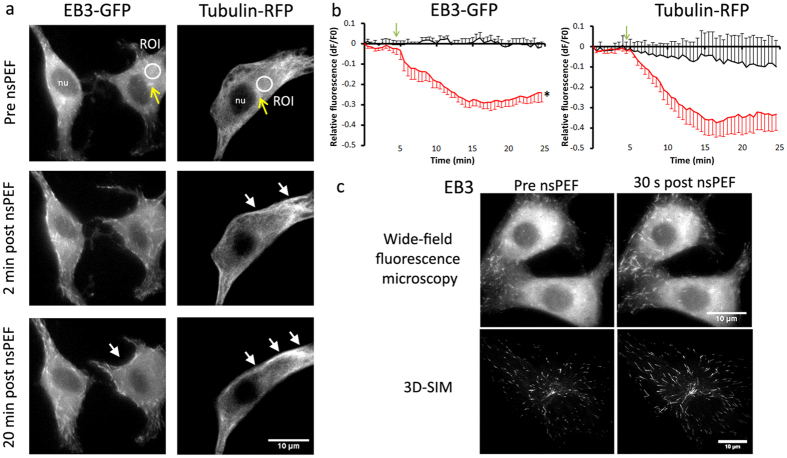
100, 10 ns pulses applied at a frequency of 10 Hz induces clearance of the microtubule network and disrupts microtubule growth. (**a**) representative live cell images of U87-EB3-GFP-tubulin-RFP cells before and 2 minutes after the application of 100, 10 ns pulses delivered at a frequency of 10 Hz. The white circle represents the location of the ROI used to measure fluorescence for Fig. 3b. White arrows denote membrane accumulation of tubulin and EB3 and yellow arrows the microtubule organising centres. (**b**) The time course of microtubule fluorescence over time plotted from live cell imaging of U87-EB3-GFP-tubulin-RFP cells. EB3-GFP (left) and tubulin-RFP (right), control cells (EB3 n = 7, tubulin n = 5) shown by the black line and nsPEF treated (EB3 n = 6, tubulin n = 4) shown in red. The start of pulse application (100, 10 ns pulses at 10 Hz) is represented by an arrow. Asterisk indicates a significant difference between groups, measured using a two-way repeated measures ANOVA [F(3.69, 40.58) = 27.17, p < 0.05, η_p_^2^ = 0.7]. Whereas a large variability in response in the tubulin conditions resulted in no differences between group means, at 5 minutes post-nsPEF post-hoc testing showed there were significant differences between the EB3 pulsed (X = −0.15, SE = 0.02) and the EB3 control conditions (X = 0.01, SE = 0.02) p < 0.05, r^2^ = 0.85. Error bars show S.E. (**c**) EB3 comets pre-pulse (left) and 30 seconds post-pulse application (right) captured with wide-field fluorescent microscopy (top) or 3D-SIM (bottom).

To test our hypothesis that microtubules will be affected by nsPEF treatment we next applied our 100 pulse at 10 Hz dosing strategy to U87-EB3-GFP-tubulin-RFP cells. Images of both the RFP and GFP channels were captured every 30 seconds during a 25 minute period with pulses applied after a baseline measurement of 4.5 minutes. Changes in fluorescence were measured in a region of interest (ROI) drawn proximal to the microtubule organising centre (Fig. 2a, circle ROI). Control experiments, where electrodes were placed but no pulse was administered, showed stable measurements of both EB3-GFP and tubulin-RFP fluorescence levels throughout the imaging period (Fig. 2b). Following pulse application a decrease in both EB3-GFP and tubulin-RFP fluorescence was observed when compared to controls not receiving nsPEFs, with a similar decrease observed when the ROI covered the whole cell (see Supplementary Figures). We interpret this decrease in tubulin-RFP fluorescence as corresponding to a breakdown of the microtubule network with an associated reduction in the number of nucleating and growing microtubules, as demonstrated by the reduction in EB3 fluorescence. Microtubule clearance was most pronounced in the cytosolic region of the cell with a loss of the fine microtubule structure and a reduction in the presence of EB3 comets, which did not recover during the imaging period. Both EB3 and tubulin showed a post-pulse accumulation near the plasma membrane (Fig. 2a, arrows). Whilst there was a reduction in EB3 fluorescence, corresponding to a reduction in the overall number of EB3 comets, the comets that remained post-pulse had an increased length (Fig. 2c). Using 3D SIM images of sufficient resolution to quantify this size change, 60 seconds after pulse application EB3 comets were 47.8 ± 4.4% (n = 11) longer than the pre-pulse comets compared to non-pulsed controls which had a 4.1 ± 3.0% (n = 4) decrease in size over the same time period (mean ± S.E.). This size difference was significant; t (13) = 6.79, p < 0.0001.

### nsPEF induced changes to microtubule growth are independent of nsPEF induced increases in intracellular Ca^2+^ levels

**Figure 3:**
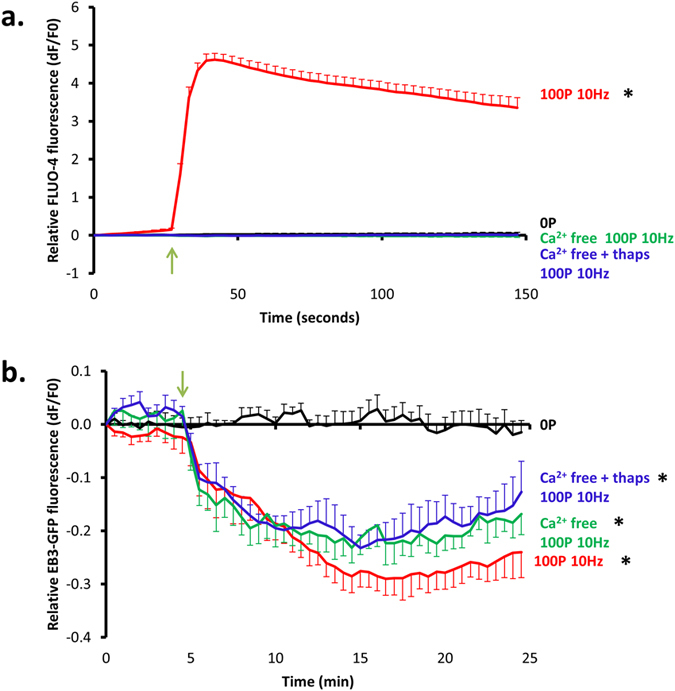
nsPEF induced disruption of microtubule growth is Ca^2+^-independent. (**a**) Change in FLUO-4 fluorescence in U87 cells subjected to 100 pulses delivered at 10 Hz. Black = non-pulsed controls (n = 9), red = pulsed Ca^2+^ HBSS (n = 11), green = pulsed Ca^2+^ free HBSS (n = 10) and blue = pulsed Ca^2+^ free HBSS with thapsigargin treatment (n = 11). A two-way repeated measures ANOVA revealed FLUO-4 fluorescence was significantly different between conditions [F(5.60, 69.10) = 262.55, p < 0.05, η_p_^2^ = 0.73]. Post-hoc testing showed that, immediately after nsPEF, the FLUO-4 fluorescence was significantly higher in the pulsed Ca^2+^ HBSS (X = 1.79, SE = 0.09) when compared to non-pulsed controls (X = 0.01, SE = 0.10), Ca^2+^ free HBSS (X = −0.01, SE = 0.10) and Ca^2+^ free HBSS with thapsigargin treatment conditions (X = −0.001, SE = 0.09), p < 0.05, r^2^ = 0.94, 0.95, 0.95, respectively. Conversely, no significant differences were found between calcium-free conditions and the non-pulsed controls. b. 100, 10 ns pulses delivered at a frequency of 10 Hz were applied to U87-EB3-GFP cells. Black = non-pulsed controls (n = 7), red = pulsed Ca^2+^ HBSS (n = 6), green = pulsed Ca^2+^ free HBSS (n = 4) and blue = pulsed Ca^2+^ free HBSS with thapsigargin treatment (n = 5). Analysis with a two-way repeated measures ANOVA showed significant differences in fluorescence between conditions [F(11.70, 81.91) = 17.30, p < 0.05, η_p_^2^ = 0.71]. Post-hoc tests showed that 3 minutes post-nsPEF exposure a significant difference in fluorescence between pulsed Ca^2+^ HBSS (X = −0.15, SE = 0.02), pulsed Ca^2+^ free HBSS (X = −0.18, SE = 0.02) and pulsed Ca^2+^ free HBSS with thapsigargin treatment conditions (X = −0.17, SE = 0.02) when compared to control conditions (X = 0.01, SE = 0.02), p < 0.05, r^2^ = 0.85, 0.90, 0.89, respectively. No significant differences were observed between treatment conditions. Asterisks indicate a significant difference when compared to control. In both cases for control experiments electrodes were positioned but no pulse was administered. The green arrow denotes the start of pulse application and error bars show S.E.

Given that one of the widely reported cellular effects of nsPEF is an increase in intracellular calcium concentration^15^,^16^,^17^ and that calcium ions are known to destabilise microtubules^28^, we next investigated whether the microtubule network disruption caused by nsPEFs was calcium-dependent. To verify that our pulsing regime caused changes in intracellular calcium concentration, we pulsed U87 cells loaded with the calcium indicator FLUO-4 AM. A 5 fold rise in FLUO-4 fluorescence, representing a significant increase in intracellular calcium concentration, was observed in the first 3 seconds following the start of pulse application. The maximal change in FLUO-4 fluorescence was reached within 15 seconds of the pulse application and levels remained elevated throughout the rest of the imaging period (Fig. 3a).

We then carried out pulse experiments on U87-EB3-GFP cells in Ca^2+^ free HBSS and in Ca^2+^ free HBSS in cells with intracellular calcium stores depleted by pre-treatment with thapsigargin (Fig. 3b). We found that removal of extracellular Ca^2+^ or extra- and intracellular Ca^2+^ had no effect on the decrease in EB3-GFP fluorescence that followed pulse application, suggesting that the effect was independent of intracellular Ca^2+^. By performing experiments on U87 cells loaded with FLUO-4 we confirmed the absence of post-pulse increases in intracellular Ca^2+^ in these conditions (Fig. 3a). Changes in EB3 comet length were also observed in both of these Ca^2+^ free conditions (see Supplementary Figures).

### nsPEF does not cause cellular swelling in U87 cells

**Figure 4:**
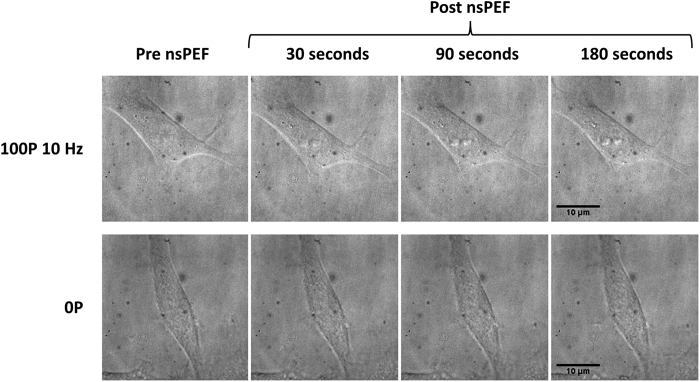
U87 cells do not swell following the application of 100, 10 ns pulses at 10 Hz. Representative phase contrast images of U87 cells before and following the application of 100, 10 ns pulses delivered at a frequency of 10 Hz (top) or non-nsPEF treated controls taken at the same time points (bottom).

Osmotic cellular swelling as a result of nsPEF induced membrane poration is a potential mechanism for the observed disruption of the microtubule network. To see if our pulse regime induced swelling in U87 cells, we observed cells following nsPEF application using phase contrast microscopy (Fig. 4). No morphological indications of swelling were observed with an absence of blebbing in all cases. An absence of swelling was also found when looking at cell area. Cells had a pre-pulse cell area of 202.1 ± 13.4 μm^2^ (n = 12). When compared to the pre-pulse cell area nsPEF treated cells showed a 3.5 ± 1.4% decrease in area at 30 seconds post nsPEF, a 6.6 ± 3.5% decrease at 180 seconds post nsPEF and a 8.1 ± 5.4% decrease 10 minutes post pulse (n = 6). Non-nsPEF treated cells at the same time points showed a 2.0 ± 2.7% decrease for 30 seconds post, a 3.7 ± 1.7% decrease for 180 seconds post and a 3.6 ± 3.6% increase at 10 minutes post (n = 6). Using a two tailed independent samples t-test, there was no significant difference in percentage volume change between cells in the control condition (X = 3.55, SE = 3.59) and cells in the nsPEF condition at the 10 minute time point (X = −8.07, SE = 5.43; t (9) = −1.78, p = 0.11). Lack of swelling was further confirmed by looking at maximally projected xz projections of U87 cells expressing Tubulin-mEmerald and imaged using 3D-SIM (see Supplementary Figures).

### nsPEFs cause microtubule buckling and breaking

**Figure 5:**
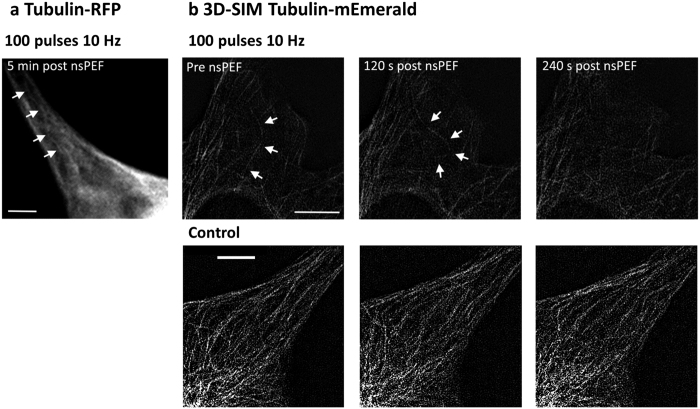
nsPEF application can cause microtubule buckling and depolymerisation. (**a**) A U87-tubulin-RFP cell imaged with wide-field fluorescence microscopy showing post-pulse buckling of a microtubule bundle, as indicated by the arrows. Scale bar = 5 μm (**b**) Top panel showing a U87 tubulin-mEmerald cell, imaged using 3D-SIM, demonstrating how individual microtubules can buckle following the pulse (120 s post pulse image) resulting in loss of the microtubule by depolymerisation (240 s post pulse image), with a time point matched non-nsPEF treated control in the bottom panel. Scale bars = 5 μm.

Post-pulse microtubule buckling was observed in several of the U87 tubulin-RFP cells, an example is shown in Fig. 5a. To see if this bending resulted in microtubule breakage we performed experiments using another imaging technique, 3D-SIM, that offered better spatial resolution at the level of individual microtubules. In U87 cells expressing tubulin-mEmerald, the application of 100 pulses at 10 Hz to U87 cells could be seen to be associated with buckling and microtubule breakage, as well as depolymerisation (Fig. 5b). Buckling and microtubule depolymerisation was not observed in control cells (Fig. 5b).

### Post-pulse microtubule disruption and loss of mitochondrial membrane are temporally correlated

**Figure 6:**
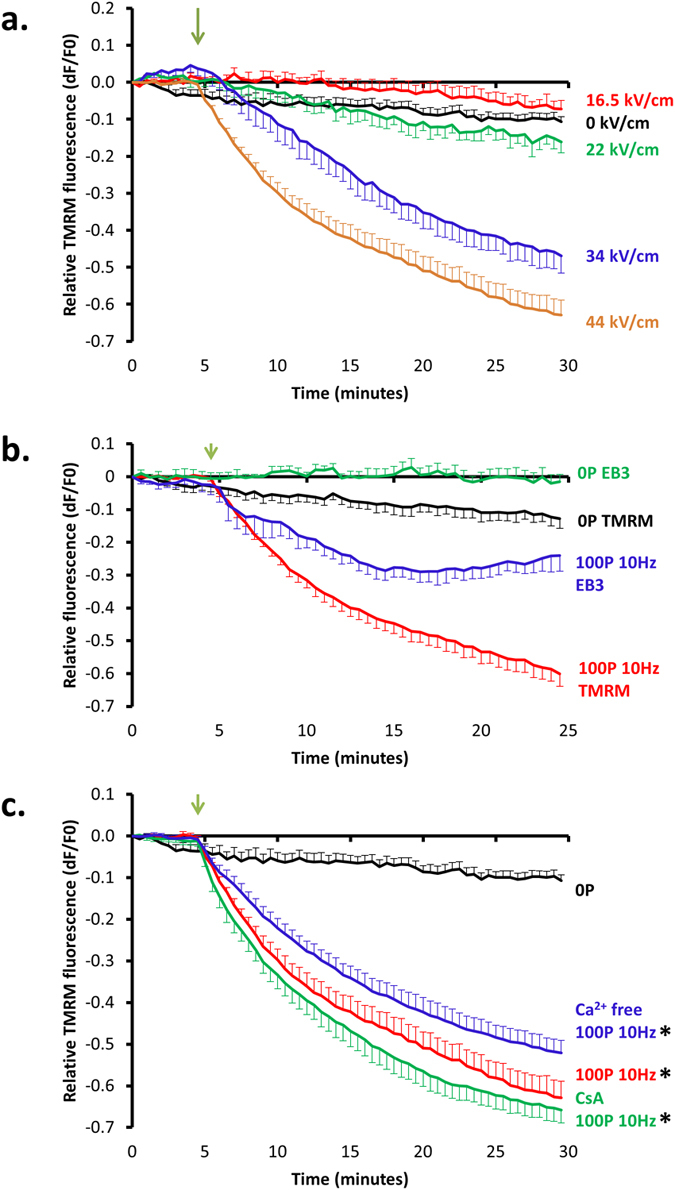
Loss of ΔΨ_m_ is temporally correlated with microtubule disruption and not due to mPTP opening. (**a**) 100, 10 ns pulses delivered at 10 Hz with electric field strengths of 0 kV/cm (black line, n = 8), 16.5 kV/cm (red line, n = 3), 22 kV/cm (green line, n = 3), 34 kV/cm (blue line, n = 4) and 44 kV/cm (orange line, n = 8) were applied to U87 cells loaded with TMRM. (**b**) 100, 10 ns, 44 kV/cm pulses were applied at 10 Hz to either U87-EB3-GFP cells (blue line, n = 6) or U87 cells loaded with TMRM (red line, n = 8) with corresponding non-pulsed controls (green line, n = 7 and black line, n = 8, respectively). (**c**) 100, 10 ns pulses delivered at 10 Hz to TMRM-loaded U87 cells in either Ca^2+^ HBSS (red line, n = 8), Ca^2+^ free HBSS (blue line, n = 8) or Ca^2+^ HBSS with pre-treatment in 10 μM cyclosporin A (CsA) (green line, n = 8) and non-pulsed control (black line, n = 8). A two-way repeated measures ANOVA showed a significant difference in fluorescence between groups [F(4.54, 42.42) = 49.31, p < 0.05, η_p_^2^ = 0.84]. Post hoc analyses at 5 minutes post-pulse revealed Ca^2+^ HBSS (X = −0.46, SE = 0.03), Ca^2+^ free HBSS (X = −0.51, SE = 0.03), and Ca^2+^ HBSS CsA (X = −0.38, SE = 0.04) conditions as being significantly different from the control (X = −0.07, SE = 0.03), p < 0.05, r^2^ = 0.91, 0.93, 0.86, respectively. No differences were measured between nsPEF conditions. Asterisks indicate a significant difference between pulsed conditions and controls. In all figures green arrows indicate the start of pulse and error bars S.E.

Chemically induced microtubule depolymerisation has previously been shown to cause loss of ΔΨ_m_ due to mechanical opening of mitochondrial permeability transition pores (mPTP)^29^. As nsPEF treatment is also known to cause loss of ΔΨ_m_ we investigated whether the post-pulse breakdown of the microtubule network caused mPTP opening leading to ΔΨ_m_ dissipation. We first determined that nsPEF treatment caused ΔΨ_m_ depolarisation in U87 cells loaded with TMRM and found an electric field strength related dose-response (Fig. 6a and Supplementary Figure). As electric field strengths increased over 22 kV/cm a greater and more rapid ΔΨ_m_ depolarisation was observed. However, at all field strengths tested no sudden and complete loss of ΔΨ_m_ was observed. We next looked for a temporal link between post-pulse breakdown of the microtubule network and ΔΨ_m_ dissipation and found that the initial decrease in TMRM fluorescence closely followed that of the disruption of microtubule dynamics, as measured by the decrease in EB3 fluorescence in U87-GFP-EB3-RFP-tubulin cells (Fig. 6b). To see if the loss of ΔΨ_m_ was due to the opening of mPTP we applied nsPEF to cells in Ca^2+^ free HBSS or to cells pre-treated with 10 μM cyclosporin A, two conditions that inhibit mPTP opening (Fig. 6c). We did not, however, find any difference in the response of the two inhibitory conditions when compared to the normal condition.

## Discussion

In this study we have shown that applying nsPEF to the human glioblastoma U87 cell line results in poration of the plasma membrane followed by breakdown of the interphase microtubule network and a concomitant reduction in number of nucleation events with a transient increase in the length of EB3 comets. We have demonstrated that the observed effects of nsPEF on microtubules are independent of post-pulse increases in intracellular Ca^2+^ concentration and cellular swelling. We also showed that the post-pulse mitochondria membrane depolarisation was gradual, temporally linked to disruption of microtubule growth and not due to influx of Ca^2+^ or opening of mPTP.

We confirmed that U87 cells undergo plasma membrane permeabilisation following the delivery of nsPEF and that, as previously reported, the extent and rapidity of poration increases with increasing pulse numbers^18^ and application frequency^30^. Using live-cell imaging we demonstrated that permeabilisation does not occur across the entire cell membrane, as YO-PRO-1 showed a polar entry and then diffused across the cell, in agreement with a previous study using 600 ns pulses^31^.

nsPEF has previously been demonstrated to cause depolymerisation of the microtubule network in both plant^32^ and mammalian cells^27^. Here we show for the first time that nsPEF causes a breakdown of the microtubule network in human cancer cells, this is important as microtubule isotype composition and post-translational modifications are often altered in cancerous cells^33^ which can effect microtubule rigidity^34^ and stability^35^. The authors of the mammalian study concluded that the microtubule depolymerisation they observed was due to increases in intracellular Ca^2+^ concentration following pulse application, as Ca^2+^ is well known to induce microtubule instability. Our results, using a different pulse regime, indicate that the initial depolymerisation observed was not mediated by changes in Ca^2+^ concentration. We did however observe a significant increase in intracellular Ca^2+^ following pulse application and, whilst our results were found to be independent of this, it is likely that under normal conditions, such elevations in Ca^2+^ would cause additional disruption to the microtubule network and other downstream signalling effects. The plant study concluded that the pulse acted directly on a membrane localised signalling hub comprised of kinesin and phospholipase D that cross-linked microtubules and actin filaments. A direct action on the membrane, resulting in microtubule disruption, cannot be ruled out in our study. nsPEF are known to affect the plasma membrane by causing poration^3^,^9^,^10^,^11^,^12^, phosphatidylserine externalisation^6^,^13^,^14^ and activation of extrinsic apoptosis^36^. Microtubules form many interactions with the plasma membrane including certain transient receptor potential channels^37^,^38^, septins^39^ and metabotropic glutamate receptors^40^, nsPEF induced membrane trauma could cause a disruption of these interactions and a resulting loss in microtubule stability.

It is possible that nsPEFs activate other lipid signalling pathways in the plasma membrane of U87 cells that influence microtubules. Mammalian cells treated with nsPEF exhibited muscarinic receptor-like signalling with phosphatidylinositol-4,5-bisphosphate (PIP_2_) depletion and calcium independent accumulation of diacylglycerol (DAG)^41^,^42^, these effects are proposed to be a result of a direct effect of nsPEF on the plasma membrane and membrane bound proteins. Agonist activation of the muscarinic receptor causes microtubule depolymerisation with translocation of tubulin to the plasma membrane, within minutes of activation, where it binds to, and inhibits the activity of, phospholipase D^43^,^44^. In the same time-scale we observed a similar membrane accumulation, of both tubulin and EB3 following pulse application. Osmotic swelling has been widely reported as a result of nsPEF^12^,^20^,^21^ however, U87 cells failed to show any signs of post-pulse swelling and tended instead to show a small decrease in cell size. This could potentially be explained by nsPEF induced activation of the PIP_2_/ inositol-1,4,5-trisphosphate (IP3) pathway which in glioblastoma plays a role in cell volume regulation. Glioblastoma cells metastasise through the extracellular space in the brain, as such they have to adapt to the tight spatial limitations of this area. They achieve this by being able to dynamically adjust their cellular volume and are able to shrink by up to 35%^45^. Cell volume change can be a result of IP3 activation by the chemotactic peptide Bradykinin, leading to the subsequent activation of outward pumping chloride and potassium channels, with cytosolic water following via aquaporins^46^,^47^,^48^. Further experiments would be necessary to confirm a role of the PIP_2_ pathway in the cell volume regulation and microtubule disruption that we observe.

Our results, however, also suggest the possibility of a direct effect of nanosecond pulsed electric fields on microtubules, as seen by their buckling and breaking and eventual network disruption. Whilst microtubules are generally rigid structures, their buckling and breaking has been previously shown to occur as a natural phenomenon in human fibroblast cells^49^. We propose that nsPEFs may cause bending by either promoting the dissociation of MAPs (proteins that bind to and stabilise microtubules), the dissociation of tubulin dimers or promoting the binding of microtubule severing enzymes. By increasing the amount of buckling microtubules within the cell, pulse application could therefore increase the amount of microtubule depolymerisation.

By imaging EB3-GFP, a protein which binds only to the tips of growing microtubules, we present novel data that provides information on microtubule dynamics following nsPEF treatment. The overall reduction in EB3-GFP fluorescence indicates that there is a decrease in microtubule nucleation, polymerisation and an increase in catastrophe. This would result in an overall decrease in the number of microtubules, which was also observed by the reduction of tubulin-RFP fluorescence signal following pulse application. An increase in EB3 comet size has been shown to occur with increased microtubule growth rate^50^, suggesting that whilst we observe an overall decrease in the number of nucleating and polymerising microtubules, those that are polymerising do so at a faster rate following the pulse treatment. The faster growth rate can be attributed to either the higher concentration of available free tubulin^50^ or to cellular swelling^51^. Given that we have shown an absence of cellular swelling it seems likely that more free tubulin is present as a result of microtubule depolymerisation.

Applying our 100 pulse, 10 Hz regime at different electric field strengths caused a dose-dependent loss of ΔΨ_m_. Even at the highest electric field intensity investigated (44 kV/cm), we still observed a gradual loss of ΔΨ_m_, rather than the rapid depolarization one would expect with pulse-induced electroporation of the inner mitochondrial membrane^18^. An alternative explanation for the loss of ΔΨ_m_ is mPTP opening which has been proposed as a downstream effect of both nsPEF treatment^19^ and chemical depolymerisation of microtubules^29^. Opening of the mPTP is triggered by increased intracellular Ca^2+ ^52 and catalysed by the binding of cyclophilin-D^53^, however, chelation of Ca^2+^ with EGTA and inhibition of cyclophilin-D with cyclosporine did not prevent the loss of ΔΨ_m_. Whilst it therefore seems unlikely that the mPTP is involved, other mitochondrial pores or channels may be implicated. One potentially interesting candidate that should be ruled out in future studies are the voltage-dependant anion channels (VDAC) which are blocked by high levels of free tubulin leading to decreased ΔΨ_m_^54^,^55^. Whilst we observed a temporal link between loss of ΔΨ_m_ and microtubule disruption, we cannot rule out the possibility that mitochondria are affected directly by the pulse or as a downstream effect of another nsPEF induced cellular event.

The data reported here suggest that nsPEF disrupt the microtubule network and affect microtubule growth by a mechanism that is independent of both calcium and osmotic swelling. Whilst this mechanism remains to be confirmed, and could be due to plasma membrane damage, increased microtubule buckling resulting in depolymerisation is a candidate. These effects might also be mediated by the electrical and/or mechanical properties of microtubules. Microtubules are dielectric structures with dipole moments that have been assessed by a number of methods, including electroorientation and simulation^56^,^57^,^58^. The resulting microtubule breakage associated with nsPEFs could therefore be caused directly by the force of the applied electric field, disrupting the electrostatic interactions of the microtubules proteins. It has already been proposed by Havelka *et al*.^59^ that the electric fields associated with nsPEFs might be sufficient to directly disrupt microtubules and this hypothesis should be tested in future *in vitro* studies. Microtubules also have mechanical properties and can be disrupted by ultrasound and shockwaves^60^,^61^,^62^. Given that nsPEFs have recently been shown to generate mechanical pressure waves^63^, it is possible that this component of the stimulus might also disrupt microtubules in exposed cells. Future experiments should therefore endeavour to distinguish the relative roles of electrical and mechanical stimuli in the observed microtubule depolymerisation associated with nsPEF exposure.

Regardless of the mechanism, a major cellular event such as the sudden breakdown of the microtubule network will likely result in numerous downstream events which could include loss of mitochondrial viability resulting in apoptosis and other effects that are desired in the treatment of cancers such as glioblastoma.

## Material and Methods

### Cell culture

The human glioblastoma cell line U87-MG (ECACC, Public Health England; 89081402) and U87 stably transfected with EB3-GFP and tubulin-RFP (U87-EB3-GFP-tubulin-RFP) were cultured in T75 flasks at 37 °C, 5% CO_2_ in MEM medium (Gibco) supplemented with 10% FBS (Gibco), 2 mM L-glutamine (Dutscher), 1.1 mM glucose (Invitrogen), 100 U/ml penicillin and 100 μg/ml streptomycin (Gibco). On reaching 80% confluence, cells were detached from the surface of the flask by washing twice with PBS (Gibco) and then incubating for 3–5 minutes at 37 °C with trypsin (PAN Biotech). Trypsin activity was stopped with the addition of an equal quantity of defined trypsin inhibitor (Gibco) and then centrifuged for 10 minutes at 600g. The resulting cell pellet was resuspended in MEM for use in experiments and for reseeding flasks.

### U87 transfection

U87 cells were transfected using LentiBrite™ EB3-GFP and LentiBrite™ RFP-Tubulin Lentiviral Biosensors (Merck Millipore). U87 cells were seeded into a 35 mm sterile dish at a density that gave 70% confluence after an overnight culture. The day following seeding the medium was replaced with fresh medium containing both lentiviruses, each at a concentration that gave a multiplicity of infection of 20. After 24 hours incubation at 37 °C, 5% CO_2_ the medium was replaced with fresh medium. On reaching confluence cells were transferred to, and maintained in, a T75 flask.

### Preparation of cells for live cell imaging

Cells were plated at a density of 1.8 × 10^5^ cells/ml into 35 mm dishes, each containing a 22 mm poly-L-lysine (Sigma) coated glass coverslip, and incubated overnight. Imaging coverslips were removed from their dishes, sandwiched into plastic imaging chambers and covered in 1 ml of room temperature HEPES-buffered salt solution (HBSS) (NaCl 121 mM, KCl 5.4 mM, MgCl_2_ 0.8 mM, NaHCO_3_ 6 mM, D-glucose 5.5 mM, HEPES 25 mM, with either CaCl_2_ 1.8 mM (Ca^2+^ HBSS) or EGTA 4 mM (Ca^2+^ free HBSS), pH 7.3). For measurement of intracellular calcium concentration, U87 cells were incubated for 30 minutes at room temperature, in the dark, in HBSS (Ca^2+^ or Ca^2+^ free) containing 0.5 μM FLUO-4, AM (Life Technologies), 0.02% pluronic acid (Life Technologies), then washed three times in the corresponding HBSS and incubated a further 30 minutes at room temperature. For measurement of cell membrane poration 1 μM of YO-PRO-1 (Life Technologies) in Ca^2+^ HBSS was added just before imaging. To deplete intracellular calcium stores cells were incubated with 1 μM of thapsigargin (Sigma) in Ca^2+^ free HBSS for 30 minute before imaging. Mitochondrial membrane potential (ΔΨ_m_) was measured by incubating cells with 10 nM Tetramethylrhodamine, methyl ester (TMRM) (Interchim) at room temperature, in the dark for 30 minutes, and washing three times in HBSS before imaging.

### Live cell imaging of U87 and U87-EB3-GFP-tubulin-RFP

Cells were observed by epifluorescence using a Leica DMI6000 microscope with a 100x objective. Fluorescent excitation was provided by a Spectra 7 light engine (Lumencor). Emitted light was filtered and captured on an electron-multiplying charge-coupled device camera (EMCCD Evolve 512, Roper) with 512 × 512 pixels. The system was controlled by, and images were captured with, Metafluor (version 7.8 Molecular Devices). Protocols for each experiment were first optimised to minimize photobleaching. GFP (excitation: 488 nm; emission: 473–575 nm) and RFP (excitation: 560 nm; emission: 590–640 nm) had exposure times and source intensities of 50 ms, 10% and 20 ms, 25% respectively. Cells showing good expression of both GFP and RFP were chosen for imaging. FLUO-4 (excitation: 488 nm; emission: 473–575 nm) images had an exposure time of 10 ms and 5% source intensity. Experiments with YO-PRO-1 (excitation: 488 nm; emission: 473–575 nm) used an exposure time of 35 ms and 10% source intensity. TMRM (excitation: 560 nm; emission: 590–640 nm) had an exposure time of 10 ms and 5% source intensity. In all experiments 2 × binning was used. A fresh coverslip was used for every control and every nsPEF treated experiment.

### Live cell, three dimensional structured illumination microscopy (3D-SIM)

For 3D-SIM U87 cells were transiently transfected by electroporation (Gene Pulser Xcell, Biorad) with either mNeonGreen-EB3 (Allele Biotechnology) or mEmerald-tubulin (Michael Davidson’s collection, Janelia HHMI). Following transfection, cells were plated onto poly-D-lysine coated FluoroDishes (WPI) and cultured for 24–48 hours before imaging. These fluorescent probes were used as were found to be brighter and more photostable than GFP, suiting the increased illumination required for 3D-SIM microscopy.

3D-SIM imaging experiments were performed on a custom system constructed at the Advanced Imaging Center of the Janelia Research Campus (Janelia HHMI, Ashburn, VA). The 3D-SIM microscope was based around a Zeiss AxioObserver microscope. A 488 nm laser (Sapphire 488–500, Coherent) was used for fluorescence excitation. The laser was rapidly shuttered with an acousto-optic deflector (AA Opto Electronic) and the structured illumination was accomplished with a ferroelectric spatial light modulatator (SXGA-3DM; Fourth Dimension Displays). Fluorescent emission was collected with a Zeiss C-Apochromat water immersion objective (magnification of 63×, N.A. = 1.2) and Z sections were sampled with an axial step size of 125 nm. A sCMOS camera (Orca Flash 4.0, Hamamatsu) was used to detect fluorescence after a dichromatic beam splitter (ZT405/488/561TPC-22.5deg; Chroma). All the system components were controlled with custom software written in Labview (National Instruments) and provided by the Janelia AIC. Complete details of the 3D-SIM microscope, method of structured illumination and the reconstruction algorithm have been described elsewhere^64^,^65^,^66^.

### nsPEF delivery system and dosimetry

**Figure 7:**
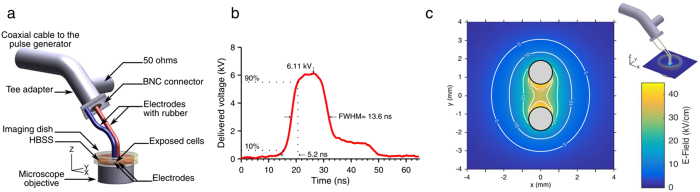
nsPEF exposure system and dosimetry. (**a**) The wire electrode delivery system used for pulse application. (**b**) Measured time-domain pulse profile for a 13.6-ns pulse FWHM duration with a 6.1 kV maximum amplitude. (**c**) Numerical simulation (FDTD) showing the spatial distribution of the electric field magnitude between the electrodes immersed in the biological sample, at the level of the coverslip, for a 6.1-kV delivered pulse.

100, 10 ns pulses, with an electric field strength of 44 kV/cm, were applied to cells at a frequency of 10 Hz using an nsPEF generator (FPG 10-1NM-T, FID Technology, Germany) with 50 Ω output impedance. A high-voltage measurement device (tap-off 245 NMFFP-100, Barth Electronics Technology, USA) connected to an oscilloscope (DPO 4104, Tektronix, USA) was used to visualize the time-domain measurements of the pulse^67^,^68^,^69^. Pulses were applied by positioning an electrode delivery system (Fig. 7a), which allowed real-time observations with the microscope objective, and comprised of two steel electrodes, separated by a gap of 1.2 mm and with 50 Ω impedance in parallel^70^. The electrodes were positioned with a micromanipulator (Sutter MP285) so that they touched the coverslip. Electric field dosimetry was calculated by finite domain-time domain method^71^ using custom software^72^.

The measured pulse profile and characteristics applied in all experiments using the nsPEF generator are shown Fig. 7b. The maximum magnitude of the delivered voltage was 6.1 kV, the pulse full width at half magnitude (FWHM) was 13.6 ns and its rise time was 5.2 ns. The nsPEF delivery system was numerically modeled using an in-house Finite Difference Time Domain (FDTD) tool providing 3D simulations of Maxwell's equations in time domain. The electric field delivered to the biological solution was assessed at the macroscopic scale at the electrode level from electromagnetic numerical simulations. Figure 7c shows the spatial distribution of the electric field obtained in the exposure area between the wire electrodes. In this central area, the electric field distribution was rather homogeneous with amplitudes of the electric field at the level of the cells in the range of 35–45 kV/cm.

### Image analysis

Image stacks from live cell imaging experiments were analysed using Image Analyst MKII (Image Analyst Software, Novato, CA). Images were first background subtracted from the stack of images by manually selecting a region of interest (ROI) in a zone that did not contain cells and subtracting these grey levels over time from in this area from the rest of the image. ROIs were then drawn within the cell (tubulin-RFP and EB3-GFP experiments), around the whole cell (FLUO-4, YO-PRO-1 experiments) or to cover the entire imaging area (TMRM experiments, see Supplementary Figures) and relative fluorescence intensity data from the ROIs were generated by Image Analyst MKII. 3D-SIM data was visualised using ImageJ (NIH). Microtubule plus-end comet size was quantified from EB3 images using Z projected 3D-SIM stacks that were thresholded and quantified with the Analyze Particles tool. Cell area was determined by drawing a free hand ROI around the perimeter of the cell and the corresponding area was calculated using ImageJ.

### Statistical analyses

Statistical analyses were performed with OriginPro 2016 software. Datasets were first tested for normal distribution using Q-Q plots and a Levene’s test was used to assess the homogeneity of variance. Changes in fluorescence intensity were statistically tested over time (repeated measures) and between independent treatments (between groups) using a mixed-model two-way repeated measures analysis of variance (ANOVA). The source of significant differences identified by ANOVAs was found using post-hoc tests with a Bonferroni correction. Effects were considered statistically significant when the probability of falsely rejection the null hypothesis was less than 0.05 (p < 0.05). For all experiments, except TMRM, n refers to a single cell separately exposed to nsPEF or control conditions. For TMRM experiments n refers to separate exposures to nsPEF or controls with all cells (generally 2–4 cells) within the imaging area monitored.

## Additional Information

**How to cite this article**: Carr, L. *et al*. Calcium-independent disruption of microtubule dynamics by nanosecond pulsed electric fields in U87 human glioblastoma cells. *Sci. Rep.* **7**, 41267; doi: 10.1038/srep41267 (2017).

**Publisher's note:** Springer Nature remains neutral with regard to jurisdictional claims in published maps and institutional affiliations.

## Supplementary Material

Supplementary Figures
