# Supplementary material for: Calcium-independent disruption of microtubule dynamics by nanosecond pulsed electric fields in U87 human glioblastoma cells
Source: Sci Rep. 2017 Jan 24;7:41267. doi: 10.1038/srep41267 (PMC5259788; doi:10.1038/srep41267)
Supplement: Supplementary Figures [file srep41267-s1.pdf]

Title

Calcium-independent disruption of microtubule dynamics by nanosecond pulsed electric fields in U87 human glioblastoma cells.

Authors

Lynn Carr[1], Sylvia M. Bardet[1], Ryan C. Burke[1], Delia Arnaud-Cormos[1], Philippe Leveque[1], Rodney P. O'Connor[2]*

*rodney.oconnor@emse.fr

1) XLIM Research Institute, UMR CNRS No 7252, University of Limoges, Faculty of Science and Techniques, 123 Avenue Albert Thomas, 87060 Limoges, France.

2) Bioelectronics Department, École Nationale Supérieure des Mines de Saint-Étienne, Centre Microélectronique de Provence - Georges Charpak Campus, 880 route de Mimet, 13541 Gardanne, France.

Supplementary figures:

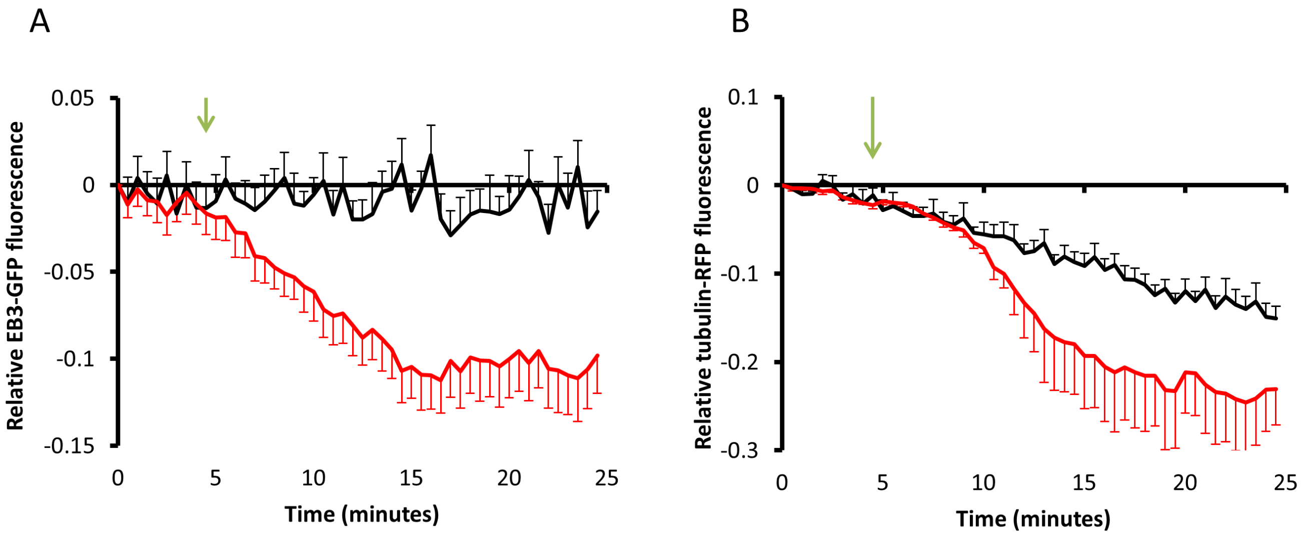

Supplementary figure 1. The time course of microtubule fluorescence over time plotted from live cell imaging of U87-EB3-GFP-tubulin-RFP cells using whole cell ROIs. EB3-GFP (A) and tubulin-RFP (B), control cells (EB3 n=7, tubulin n=5) shown by the black line and nsPEF treated (EB3 n=6, tubulin n=4) shown in red. The start of pulse application (100, 10 ns pulses at 10 Hz) is represented by an arrow.

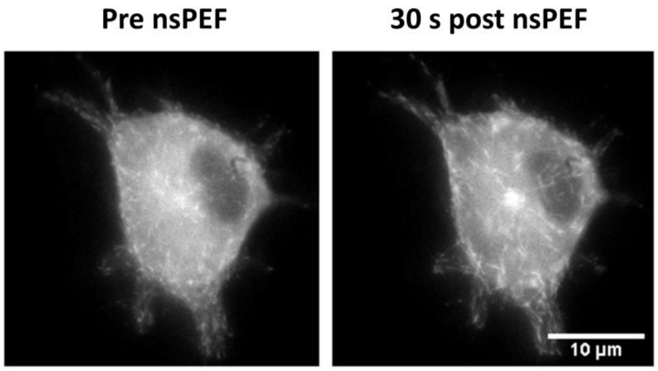

Supplementary figure 2. Representative live cell images showing EB3 comets in a U87-EB3-GFP cell before and 30 seconds after application of 100, 10 ns pulses at 10 Hz in EGTA HBSS.

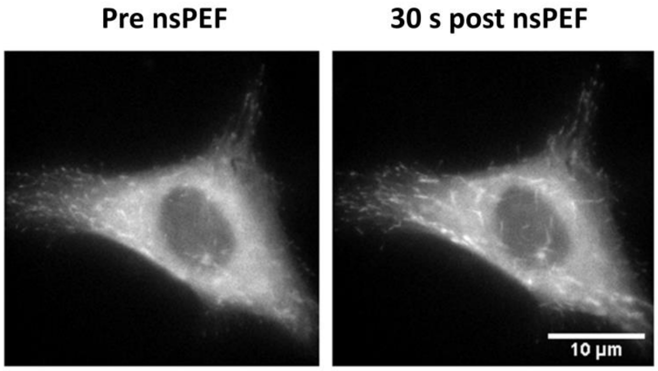

Supplementary figure 3. Representative live cell images showing EB3 comets in a U87-EB3-GFP cell, pre incubated with thapsigargin, before and 30 seconds after application of 100, 10 ns pulses at 10 Hz in EGTA HBSS.

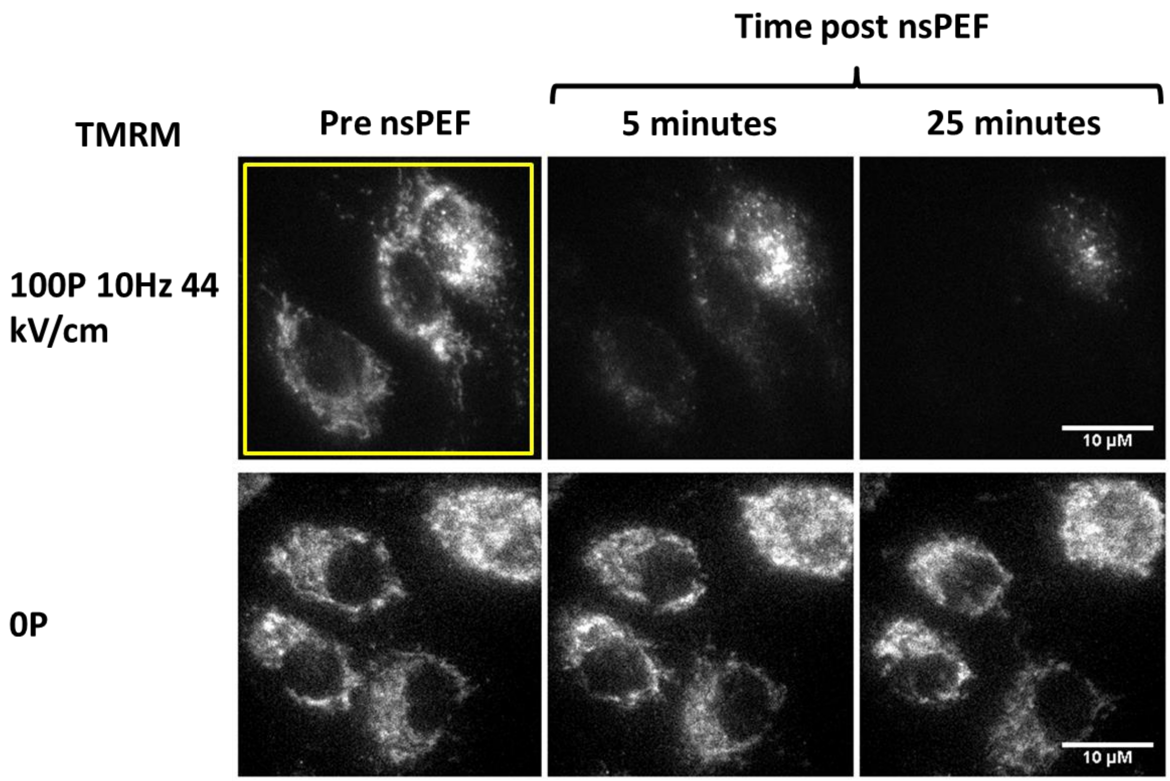

Supplementary figure 4. Representative live cell images showing U87 cells loaded with TMRM before and after application of 100, 10 ns pulses at 10 Hz (top) and control where no pulse was applied (bottom). The yellow box indicates the ROI used for measuring changes in fluorescence.

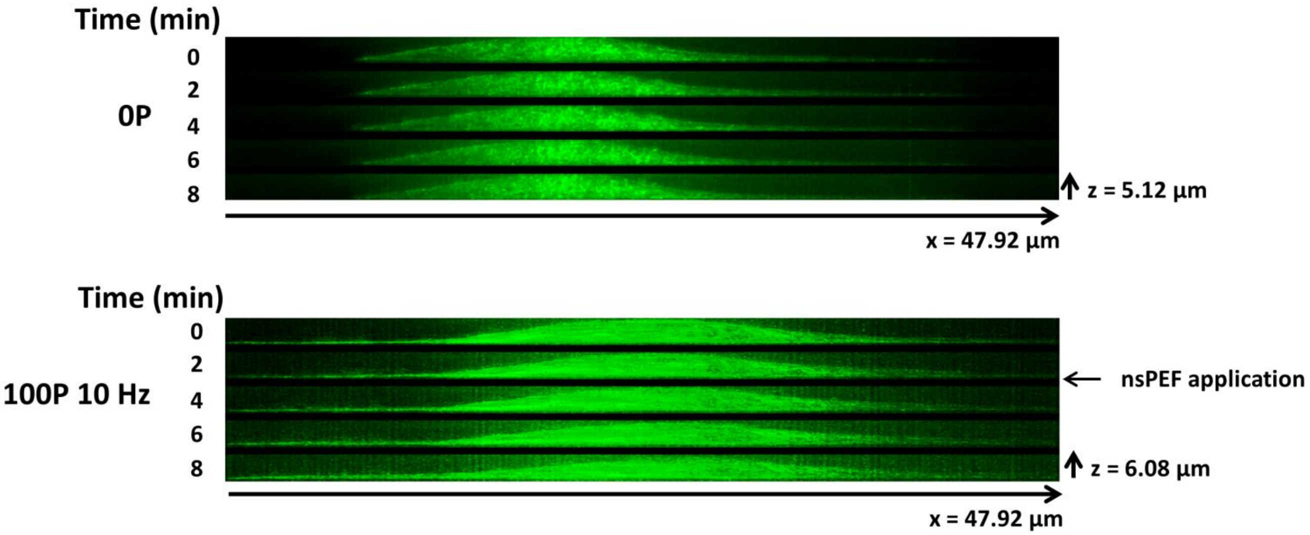

Time (min)	Surface area of side projection µm²		Change in surface area (% of t = 0)	
	0P	100P 10 Hz	0P	100P 10 Hz
0	86.41	105.57	0.00	0.00
2	90.05	105.53	4.21	-0.04
4	93.93	111.38	8.70	5.50
6	98.19	117.60	13.63	11.40
8	103.44	123.23	19.71	16.73

Supplementary figure 5. Representative, maximally projected xz projections of U87 cells expressing Tubulin-mEmerald and imaged using 3D-SIM. Cells were either exposed to 100, 10 ns pulses at 10 Hz or no pulses. The table shows the surface areas of these side projections and there % size change over the imaging period.
